# Functional validation of novel levamisole resistance marker S168T in *Haemonchus contortus*

**DOI:** 10.1016/j.ijpddr.2024.100524

**Published:** 2024-02-06

**Authors:** Alistair Antonopoulos, Claude L. Charvet, Kirsty Maitland, Stephen R. Doyle, Cédric Neveu, Roz Laing

**Affiliations:** a School of Biodiversity, One Health and Veterinary Medicine, University of Glasgow, Glasgow, Scotland, United Kingdom; b Kreavet, Kruibeke, Belgium; c INRAE, Université de Tours, ISP, F-37380, Nouzilly, France; d Wellcome Sanger Institute, Hinxton, Cambridgeshire, United Kingdom

**Keywords:** Levamisole resistance, S168T, *Haemonchus contortus*, Resistance mechanism, Functional validation, Acetylcholine receptor

## Abstract

Recently, a S168T variant in the acetylcholine receptor subunit ACR-8 was associated with levamisole resistance in the parasitic helminth *Haemonchus contortus*. Here, we used the *Xenopus laevis* oocyte expression system and two-electrode voltage-clamp electrophysiology to measure the functional impact of this S168T variant on the *H. contortus* levamisole-sensitive acetylcholine receptor, L-AChR-1.1. Expression of the ACR-8 S168T variant significantly reduced the current amplitude elicited by levamisole compared to acetylcholine, with levamisole changing from a full to partial agonist on the recombinant L-AChR. Functional validation of the S168T mutation on modulating levamisole activity at the receptor level highlights its critical importance as both a mechanism and a marker of levamisole resistance.

## Graphical abstract

**Figure:**
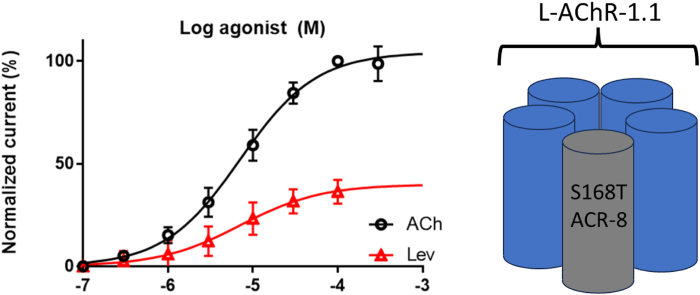

## 1 Introduction

The broad-spectrum anthelmintic levamisole (LEV) is widely used to treat veterinary helminth infections (Kotze and Prichard, 2016) and occasionally used in the treatment of human infections with *Ascaris* spp., and *Taenia* spp. (Aribodor et al., 2021). Clinical trials to assess LEV for treating loiasis are ongoing (Campillo et al., 2022). LEV is a cholinergic agonist drug which binds to nematode ligand-gated ion channels, specifically levamisole-sensitive acetylcholine receptors (L-AChRs), in the body wall muscles of the parasite (Aceves et al., 1970; Martin et al., 1997; Kopp et al., 2009). The binding of LEV causes the channel to open, which causes spastic paralysis of the parasite and leads to expulsion from the host (Martin and Robertson, 2007; Martin et al., 2012). The reconstitution of *Haemonchus contortus* L-AChRs using the *Xenopus laevis* oocyte expression system has been instrumental in characterising the composition of these pentameric L-AChRs (Fauvin et al., 2010; Neveu et al., 2010; Boulin et al., 2011; Blanchard et al., 2018). The *H. contortus* L-AChR subunits UNC-29.1, UNC-38, UNC-63, and ACR-8 assemble to form the L-AChR-1.1 subtype (Boulin et al., 2011). In contrast, the substitution of UNC-29.1 by either UNC-29.3 or UNC-29.4 will form the L-AChR-1.3 and L-AChR-1.4 subtypes, respectively (Duguet et al., 2016). Delineating the functional composition of these receptors revealed that ACR-8 is crucial in conferring sensitivity to LEV *in vitro* and *in vivo* (Boulin et al., 2011; Blanchard et al., 2018). Indeed, in the absence of ACR-8, the combination of UNC-29.1, UNC-38, and UNC-63 will assemble to form Hco-L-AChR-2, a receptor subtype relatively unresponsive to levamisole but highly sensitive to nicotine and pyrantel (Martin et al., 2012).

Recently, a forward genetic cross between multidrug-resistant and drug-susceptible strains of *H. contortus* revealed a non-synonymous single nucleotide polymorphism (SNP) in *acr-*8 that was strongly associated with LEV resistance (Doyle et al., 2022). This SNP conferred a serine-to-threonine substitution (S168T) in exon 4, which was present in all LEV-resistant populations examined to date (including USA, South Africa, and Australia) and absent in all sensitive populations with available sequencing data (Sallé et al., 2019; Antonopoulos et al., 2022; Francis et al., 2024). Furthermore, a serine-to-threonine substitution was present at the analogous position of *acr-8* in LEV-resistant *T. circumcincta* after re-analysis of existing data (Choi et al., 2017; Doyle et al., 2022). This putative convergent evolution would suggest that T168 may play a mechanistic role in LEV resistance in different trichostrongylid species. While S168T represents a robust genetic marker for LEV resistance, the functional relevance of this mutation remained undefined. Here, we have used the *X*. *laevis* heterologous expression system to recapitulate the *H. contortus* L-AChR and measure the functional impact of S168T on the LEV receptor target.

## 2 Materials and methods

### 2.1 Cloning the *acr-8* S168T variant

Primer sequences to amplify the entire coding sequence of the *H. contortus acr-8* gene (HCON_00151270) (Doyle et al., 2022) were designed in Geneious Prime (Biomatters Ltd: 11.1.5) and ordered from Eurofins Genomics: acr8F (ATGCGTGCATTCGGAATTG) and acr8R (TCACAAGCCTTCAGAATTC). Total RNA was extracted from a pool of 20 *H. contortus* adult males and females of the multi-drug (levamisole, macrocyclic lactone, and benzimidazole) resistant MHco18 (UGA2004) isolate and used for cDNA synthesis following standard methods previously described (Doyle et al., 2022). Phusion Green High-Fidelity PCR was carried out according to manufacturer instructions to amplify the full-length *acr-8* gene with the following parameters: 40 cycles: denaturation at 95 °C for 30 s, annealing 58 °C for 30 s, and extension at 72 °C for 120 s, with a final extension at 72 °C for 10 min. The resulting *acr-8* amplicon was cloned into the TOPO2.1 vector (Thermo Fisher) and transformed into XL10 gold ultracompetent *E. coli* (Agilent), as previously described (Antonopoulos et al., 2022). Colonies were screened with an allele-specific PCR for S168T as described previously (Antonopoulos et al., 2022) before plasmid isolation and capillary sequencing at Eurofins Genomics. The full-length *acr-8* with S168T in TOPO2.1 was then subcloned into the transcription vector pTB207, which contained the 3′UTR of *X*. *laevis* beta-globin (Boulin et al., 2011). The resulting construct was sequence-checked, linearised and used as a template for *in vitro* cRNA synthesis using the mMessage mMachine T7 transcription kit (Ambion).

### 2.2 Expression of L-AChR subunits in *Xenopus* oocytes and electrophysiology

Expression of the L-AChR subunits *unc-29.1*, *unc-38*, *unc-63* and *acr-8* (wild type or S168T variant) in *X*. *laevis* oocytes (Ecocyte Bioscience) and two-voltage clamp electrophysiology manipulations were performed as previously described (Boulin et al., 2011; Blanchard et al., 2018). In brief, *X. laevis* oocytes were injected with 36 nL of cRNA mix containing 50 ng μL^−1^ of each subunit: *unc-63, unc-29.1, unc-38,* and *acr-8* (S168 or S168T variant), along with 50 ng μL^−1^ of each *H. contortus* ancillary proteins *ric-3.1, unc-50,* and *unc-74* (Boulin et al., 2011). Furthermore, a 1:1 mix of S168 and S168T (25 ng μL^−1^ of each to a total of 50 ng μL^−1^) was used along with *unc-63, unc-29.1, unc-38* and the ancillary protein cRNAs. All transcription vector constructs were generated in these previous studies, other than the construct encoding the S168T variant *acr-8* described above. Currents elicited by levamisole (L9756, Sigma-Aldrich) and acetylcholine (A9101, Sigma-Aldrich) were analysed using the pCLAMP 10.4 package (Molecular Devices). All concentration-response relationships were carried out 4–5 days after injection in recording solution consisting of 100 mM NaCl, 2.5 mM KCl, 1 mM CaCl_2_, and 5 mM HEPES, at pH 7.3 (Boulin et al., 2011). The currents are shown as the ratio of the responses to that for 100 μM ACh. The EC_50_ value is the concentration giving half of the maximum response and Imax is the relative maximal current obtained at saturating agonist concentration. Results are shown as mean ± SEM. For statistical analysis, a two-sample *t*-test (https://select-statistics.co.uk/calculators/two-sample-t-test-calculator/) was used to compare the mean recordings of each receptor against the wild-type receptor.

## 3 Results and discussion

**Fig. 1:**
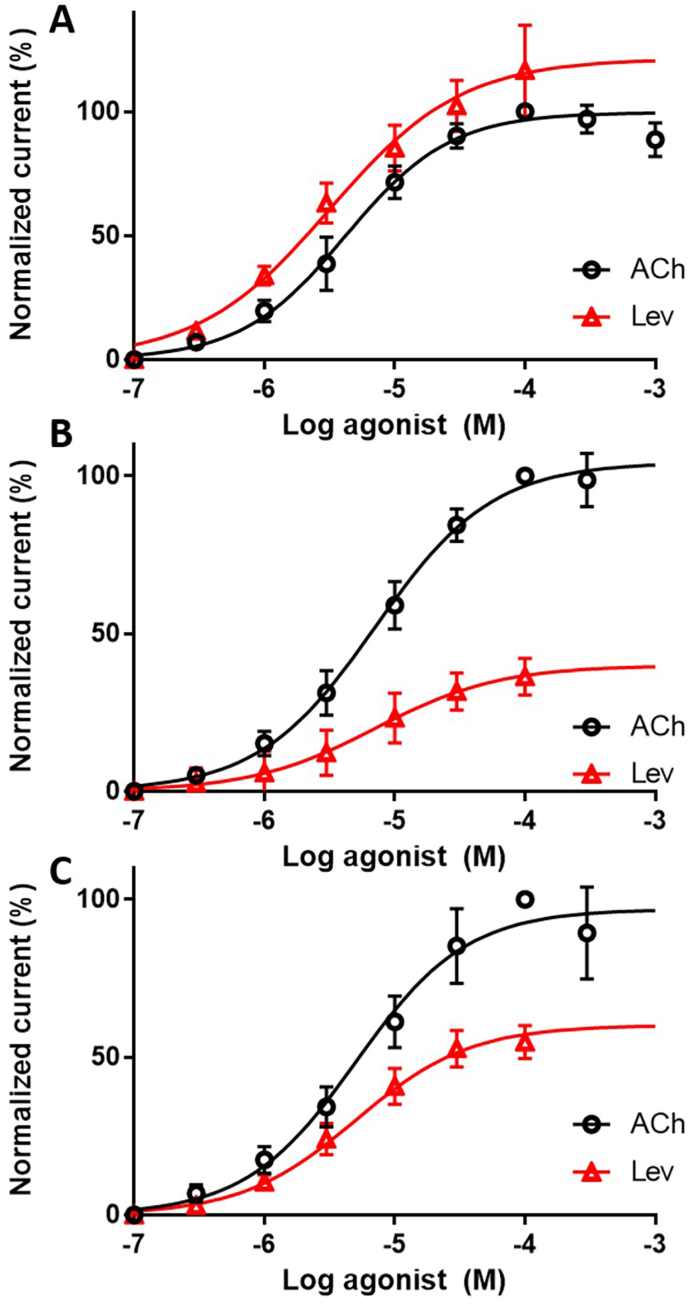
Concentration-response curves of ACh (black) and LEV (red) on the *Haemonchus contortus “*wild-type” L-AChR-1.1 receptor (A), on the L-AChR-1.1 with the S168T Hco-ACR-8 replacing the wild-type Hco-ACR-8 subunit (“homozygous mutant”) (B), and on the L-AChR-1.1 with S168T Hco-ACR-8 and wild-type Hco-ACR-8 at a 1:1 relative ratio (“heterozygous mutant”) (C). All responses were normalized to 100 μM ACh. The data shown represents mean ± SEM. Sample sizes are shown in Table 1.

To examine the functional impact of S168T on the AChR, the *H. contortus* L-AChR-1.1 was reconstituted in *Xenopus* oocytes by microinjection of the *unc-29.1*, *unc-38*, *unc-63* and *acr-*8 cRNAs and assessed with two-electrode voltage-clamp electrophysiology. The expression of *H. contortus* L-AChR-1.1 with wild-type ACR-8 produced functional channels with acetylcholine (ACh) and LEV EC_50_ values of 4.2 ± 1.1 (n = 16) and 3.2 ± 1.1 μM (n = 15), respectively. These findings were consistent with previous studies, with LEV acting as a super-agonist (121.4 ± 3.3 % of ACh response) (Fig. 1A, Table 1) (Boulin et al., 2011; Blanchard et al., 2018) (see Table 2).

**Table 1:** Summary of the EC_50_ and I_max_ values for acetylcholine (ACh) and levamisole (LEV) on the Hco-L-AChR-1.1 and the different ACR-8 subunits assessed in the context of L-AChR-1.1 expressed in *Xenopus* oocytes. All currents were normalized to the maximum response obtained by 100 μM ACh. I_max_ is the relative maximal current obtained at saturating agonist concentration. Results are shown as mean ± SEM.

L-AChR subunit combination		ACh EC_50_ (μM)	LEV EC_50_ (μM)	ACh I_max_	LEV I_max_
UNC-29/UNC-38/UNC-63	+ Hco-ACR-8 (“homozygous wild-type”)	4.2 ± 1.1 (n = 16)	3.2 ± 1.1 (n = 15)	99.7 ± 1.3 (n = 16)	121.4 ± 3.3 (n = 15)
	+ Hco-ACR-8 S168T (“homozygous mutant”)	7.1 ± 1.1 (n = 7)	6.8 ± 1.3 (n = 13)	104.4 ± 2.4 (n = 7)	39.5 ± 3.1 (n = 13)
	+ Hco-ACR-8+ Hco-ACR-8 S168T (“heterozygous”)	5.3 ± 1.1 (n = 7)	4.1 ± 1.1 (n = 8)	97.8 ± 3.1 (n = 7)	57.0 ± 1.7 (n = 8)

**Table 2:** Summary of statistical comparisons of EC_50_ and I_max_ values for L-AChR subunit combinations. ACh. I_max_ is the relative maximal current obtained at saturating agonist concentration.

L-AChR subunit combination (ligand) EC_50_	Statistical significance (p-value)
S168 vs S168T (ACh)	p=<0.001
S168 vs S168T (LEV)	p=<0.001
S168 vs S168 + S168T (ACh)	p = 0.048
S168 vs S168 + S168T (LEV)	p = 0.082

L-AChR subunit combination (ligand) I_max_	Statistical significance (p-value)
S168 vs S168T (ACh)	p = 0.001
S168 vs S168T (LEV)	p=<0.001
S168 vs S168 + S168T (ACh)	p = 0.162
S168 vs S168 + S168T (LEV)	p=<0.001

To assess if the S168T ACR-8 subunit could assemble into functional L-AChRs, we replaced the wild-type ACR-8 with the S168T variant (Fig. 1B); this resulted in functional channels, evidenced by large ACh-induced currents. Strikingly, the application of 100 μM LEV produced significantly smaller currents than ACh (39.5 ± 3.1 % of ACh response, p=< 0.001), indicating that LEV acts as a partial agonist on channels containing the S168T ACR-8 variant. The ACh and LEV concentration-response curves were characterised by EC_50_ values of 7.1 ± 1.1 (n = 7) and 6.8 ± 1.3 μM (n = 13), respectively, which are significantly higher than L-AChR containing wild-type ACR-8 (p=< 0.001). With LEV behaving as a partial agonist, it could be hypothesised that these results may be explained by the formation of an L-AChR-2 subtype made of UNC-29.1, UNC-38 and UNC-63 subunits only (Boulin et al., 2011). However, the fact that the ACh and LEV EC_50_ values were much closer to those of L-AChR-1.1 and were significantly lower than those of L-AChR-2 argues (19.2 ± 0.7 and 48.2 ± 0.9 μM, respectively) against this hypothesis.

To mimic a heterozygous genotype of a worm potentially harbouring mixed populations of L-AChR-1.1, we co-injected cRNAs of S168T *acr-8* along with wild-type *acr-8* at a 1:1 concentration ratio (Fig. 1C). Surprisingly, the inclusion of both variants led to a significant reduction in the average current to 57.0 ± 1.7 % of the 100 μM ACh response (p=<0.001) after exposure to 100 μM LEV. No significant difference (p = 0.082) in the LEV EC_50_ values was found between the eggs expressing both variants (4.1 ± 1.1 μM, n = 8) and eggs expressing only the wild-type-L-AChR-1.1 (3.2 ± 1.1 μM, n = 15). However, a significant difference (p = 0.048) was found between the ACh EC_50_ value of the wild-type L-AChR-1.1 (4.2 ± 1.1 μM, n = 16) and the mixed L-AChRs (5.3 ± 1.1, n = 7). This suggests that by making LEV a partial agonist, the S168T variant could confer a slightly lower susceptibility to LEV in heterozygous worms, although not to a significant degree. The lack of a significant difference observed between the EC_50_ values for LEV evoked currents in the *Xenopus* oocytes containing both the S168 and S168T variant, but a significant difference in EC_50_ values for ACh ACR-8, implies that receptor function could be compromised in heterozygous worms. However, final validation of this observation *in vivo* would require transgenesis, which is not currently feasible in *H. contortus*.

## 4 Conclusion

In summary, we demonstrate that the S168T ACR-8 subunit can substitute for wild-type ACR-8 to form a functional receptor, resulting in a change in the action of LEV from full agonist to partial agonist. This result is consistent with the lack of LEV sensitivity of the *C. elegans* ACR-8 (Qian et al., 2008; Hernando et al., 2012), which possesses a threonine at the analogous position. Further, the presence of the S168T variant at the equivalent position in LEV-resistant *T. circumcincta* ACR-8 (Choi et al., 2017) suggests this variant could be the basis for a conserved resistance mechanism in different trichostrongylid species. These data support that the S168T variant in ACR-8 underlies the LEV resistance phenotype in parasites carrying this mutation and represents a significant development toward defining the molecular and genetic basis of LEV resistance.

## Declaration of competing interest

The authors report no conflict of interest for this work.
